# A Sick Mind

**Published:** 1880-02

**Authors:** 


### A SICK MIND.

NOT a diseased mind; that might imply something akin to insanity; such is not our meaning. The body may be diseased functionally; the whole machinery is perfect as to parts, no portion is broken or destroyed, but it does not work well,—so we mean as to the mind, when we term it "sick;" it is perfect, it is sound, no decay, but it does not work smoothly, effectively.

